# Female Pakistani carers’ views on future formal and informal care for their older relatives in Norway

**DOI:** 10.1186/s12913-020-05468-z

**Published:** 2020-07-01

**Authors:** Sanjana Arora, Bernd Rechel, Astrid Bergland, Melanie Straiton, Jonas Debesay

**Affiliations:** 1 Faculty of Health Sciences, Department of Physiotherapy, Oslo Metropolitan University, P.O. Box 4, St. Olavs plass, N-0130 Oslo, Norway; 2 European Observatory on Health Systems and Policies, London School of Hygiene and Tropical Medicine, 15-17 Tavistock Place, London, WC1H 9SH UK; 3 Division of Mental and Physical Health, Norwegian Institute of Public Health, PO Box 222, Skøyen, 0213 Oslo, Norway; 4 Faculty of Health Sciences, Department of Nursing and Health Promotion, Oslo Metropolitan University, P.O. Box 4, St. Olavs plass, N-0130 Oslo, Norway

**Keywords:** Caregiving, Residential care homes, Professional homecare, Immigrants, Norway

## Abstract

**Background:**

The aging of Pakistani immigrants in Norway raises questions related to their increased need for care and help from relatives, as well as those concerning what future formal and informal care and healthcare accessibility for older immigrants may look like. The hidden nature of family caregiving means that the circumstances of carers, their views and their dilemmas related to future care are largely invisible. In this study, we explored female Pakistani carers’ views of future care and healthcare accessibility for their older relatives in Norway.

**Methods:**

Our data included interviews with family carers between the ages of 23 and 40 years old, living in Oslo, Norway. We recruited ten family carers, out of which eight were daughters and two were daughters-in-law. Interviews were conducted by the first author in Urdu or English and were recorded and transcribed verbatim.

**Results:**

Our findings revealed several factors that influenced participants’ perceptions about formal and informal caregiving, which can be organised into the following themes: 1) caring for family in Norway as in Pakistan, 2) worries about being ‘dropped off’ at a care home, 3) concerns about being cared for by outsiders, 4) questions about what other people might say and 5) adhering to society’s expectations of a ‘good’ carer.

**Conclusion:**

Family carers’ traditional views of filial piety do not entirely determine the use of or access to healthcare services of their older relatives. There is a need to develop culturally sensitive healthcare systems so that immigrant families and their carers have more options in choosing care in old age, which in turn will ease their families’ care burden. Healthcare professionals and policymakers should not assume that immigrant families will take care of their own older members but should instead secure adequate support for older immigrants and their family carers.

## Background

Demographic, socio-economic and political trends throughout high-income countries have resulted in the care of older people becoming an issue of utmost policy importance. Older people have greater healthcare needs than do the general population and are at a higher risk of adverse health outcomes [1]. Furthermore, the strength of the association between migrant status and frailty has been found to be great, particularly among those from low- or middle-income countries [2]. These groups may also underuse public care services [3–5], and thus, the well-being of older immigrants can be largely dependent on their family carers. Informal/family carers are unpaid individuals, such as family members, friends and neighbours, who provide as much as 90% of the in-home long-term care required. Many family carers spend 4 to 7 years—and as much as 15 to 20 years—doing a job that is stress-filled, overwhelming and isolating [6].

Norway, like the rest of Europe, is witnessing significant demographic changes in its immigrant population. While only 5% of immigrants were 70 years or older in 2018, this share is expected to increase to 25% by 2060 [7]. The Pakistani population in Norway constitutes one of the largest, and longest residing, groups among non-European immigrants [8]. Aging within this group therefore raises the concern of an increased need for care and help from relatives and the question of how future formal and informal care and healthcare accessibility for older immigrants could, or should, look in Norway.

### The benefits and costs of family caregiving

Research suggests that the experience of providing care may differ for immigrants and their descendants. Family caregiving can have its rewards and benefits. For example, it can lead to the appreciation of life, personal growth, enhanced self-efficacy, competence or mastery, self-esteem, and closer relationships [9–11]. Furthermore, positive psychological effects due to caregiving may mitigate some of the challenges of caregiving, as positive effects are associated with lower levels of burden and depression and better overall mental health [12]. However, family caregiving can also have invisible costs. Although most children feel responsible and are motivated to care for their parents or in-laws [13], there is uncertainty about their ability and willingness to assume full responsibility for such care. These doubts are hardly discussed within families, and most often, older immigrants do not want to be a burden to their adult children [3]. Thus, informal caregiving, including the circumstances of carers, the challenges they experience and their need for support from formal care services as well as other informal networks, is often hidden or even invisible. Along with the physical challenges of caregiving, informal caregiving can also have a negative psychological, social and emotional impact on carers, such as feelings of guilt, embarrassment, stress and anxiety [14–16]. Moreover, informal caregiving continues to be a highly gendered activity, with women performing the bulk of the family care in most cases [17]. Furthermore, chronic stress due to informal caregiving has been linked to poor health outcomes, morbidity and mortality [18]. Those born in the receiving country could face greater challenges in caregiving than do the older immigrant relatives for whom they provide care, due to having different values and/or expectations about family relationships and the expectations of older relatives more closely mirroring experiences they had in their countries of origin [19].

While there is an increasing amount of research on the health of immigrant populations in Norway [20], issues regarding ageing and care for older immigrants have received very little attention. A few studies [21, 22] and reports [23–25] have started to provide insights into the care needs of older immigrants in Norway and the availability of care services for them, but hardly any research has so far been undertaken about the views of family carers to older immigrants. Our study aims to fill some of this gap.

### Pakistani immigrant women in Norway and caregiving

The first Pakistani immigrants arrived in Norway in the late 1960s and early 1970s as labour immigrants, followed by women who migrated mainly through family reunification [26]. The issue of older Pakistani women’s care is of particular concern because of their social disadvantages in the areas of education [27], employment [27] and self-reported health [20], as compared to Pakistani men. In fact, a study on older male Pakistani immigrants in Norway reported that a few participants perceived that residential care homes might be more suitable for Pakistani men than they are for women; because the latter typically spend most of their time within the confines of the household, their social world has been limited to their family [28].

Studies have shown that the descendants of immigrants often identify as both Norwegian and Pakistani, but when it comes to caring for older people, they tend to uphold traditional values from Pakistan, as filial obligations are morally compounded in culturally implicit ‘generational contracts’ [21, 29, 30]. Moreover, upon marriage, it is common for a woman to move in with her husband’s family, where she might face certain expectations when it comes to the wellbeing of her parents-in-law [21]. Thus, caregiving continues to be a gendered activity, in particular for daughters-in-law. In this context, it becomes imperative to examine the perspectives and experiences of female carers, so that better care is not only provided to older immigrants in future but also to ensure the well-being of their carers. Thus, the aim of this article is to explore female Pakistani carers’ views on the future formal and informal care and healthcare accessibility of their older relatives in Norway.

## Methods

This article is based on qualitative interviews with family carers of older Pakistani women living in Oslo municipality, Norway. The participants were recruited through snowball sampling, starting with meeting women at a local mosque, at an activity centre and through key informants. They were followed up via phone to arrange face-to-face interviews at a later date. Our recruitment criteria for family carers were that they perceived themselves to be the primary provider of care for an older female relative or that they were primarily involved in facilitating access to formal health care by accompanying an older female relative to appointments. It should be noted that of those who identified themselves as primary carers, we only found women, despite being open to the recruitment of male carers. All participants who were approached and fulfilled the recruitment criteria agreed to participate in the study.

The family carers were between the ages of 23 and 40 years. We recruited 10 family carers, out of which 8 were daughters, all born in Norway, and 2 were daughters-in-law, of which one was born in Norway. Seven carer daughters had full-time jobs, and one was a student. Of the two carers who were daughters-in-law, one worked part-time and the other was a student. All our participants’ either had higher education or were pursuing higher education.

We developed a semi-structured interview guide to explore the following: 1) family carers’ perceptions and experiences of caring for their older mother/mother-in-law, and 2) family carers’ perceptions of residential care homes and homecare services for their relatives (see Table 1). The interviews were conducted by the first author, a PhD student who has experience of conducting research on topics related to healthcare among immigrant women in Norway and is an immigrant woman of South-Asian origin. A somewhat shared background with the participants helped to minimise the distance and the interviews were thus informal and interactional. Interview appointments were followed up via phone which also helped to build rapport prior to conducting the interviews. Nine interviews were conducted in Urdu and one interview was conducted in a mix of Urdu and English, based on the participants’ preferences. Interviews lasted 45 min to 1 h and were recorded and transcribed verbatim. They were conducted at participants’ homes or in public at cafes or parks with only the first author and participant present. Field notes were made by first author after each interview, summarizing important findings and noting reflections and suggestions for themes and patterns that were identifiable at that stage. Participants were thus probed for relevant issues that emerged. The data was analysed by thematic analysis using Braun and Clarke’s six-phase guide [31]. Specifically, we performed the following steps: familiarising ourselves with the data, generating initial codes, searching for patterns or themes across data and identifying relevant themes. In the next stage, we reviewed and named the themes (see Table 2 for reference). Nvivo was used to aid in coding.

**Table 1:** Interview guide for carers

	Questions	Themes
1.	- Can you tell me about yourself? How do you feel about living here? - How do you usually spend your day?	Warm up questions
2.	- Could you tell me about your caring role? Who do you care for? What age is the person you care for? - What are your day to day caregiving responsibilities? How do you feel about them? - How are you involved in decision making regarding health of the older person- Types of decisions made for this older person in the past.	Caregiving responsibilities and experience
3.	- How are other family members involved in Health care? - Interpreting written materials - Attend doctors/hospital - Navigating health system - Decision Making	Role of other members
4.	- Process of deciding which health care service to use to best meet the needs of the older person and when? Does your relative ever delay help seeking? If so, why? - Importance of GP/Nurse of Pakistani background	Use of health care
5.	How have you found Norwegian healthcare services? - Can you tell me about your experience with the GP when you visited with your relative last time? - Any difference in experience with GP regarding your own health matters as compared to that of your relative’s? - Do you also act as an interpreter for your relative? What are the challenges in this process? - Last experience with the GP or/and Hospital? How would you describe the quality of care and treatment that they received? - How has your experience been of accessing health care services in Pakistan for/with your relative?	Experience and Satisfaction from health care services in Norway and Pakistan
6.	- How does your relative get healthcare information? (If caregiver facilitates access to health care information. Ask further) - How do you feel about the accessibility of the Norwegian Health care services? - Difficulties while seeking/accessing health care because of difficulty understanding written and/or spoken information?	Knowledge and Awareness
7.	- Has your relative used professional care services such as nursing homes or home care? If not,: How would you and your relative feel about using it in future?	Perception about professional/formal care
8.	- What suggestions would you make to improve to health care experience of older Pakistani women?	Suggestions for improvement of health care
9.	- Is there anything else you want to add about health or health care that you’ve not had a chance to say yet?	Closure

**Table 2:** Illustration of thematic analysis

Meaning units	Condensed text/Codes	Sub-themes	Themes
*[W]hen mother was in hospital, we used to go there every day to see her and bring her food,* etc. *So the white people who were staying there long-term started telling their own stories, saying nobody comes to see them—only at Christmas or on other [holidays].*. *.*. *Then we understood that they long for this*. *.*. *for someone close to them to come and meet with them. Even doctors say that ger-mulki [immigrants] are very good at such things; they care for their elderly a lot.*	Perception that Norwegian (white) children do not care as much for their parents as the Pakistani children do. Perception that Norwegians desire for such type of care and appreciate immigrant children for doing the same.	Feeling appreciated as an immigrant for caring for their older parent by Norwegians	Adhering to society’s expectations of a ‘good’ caregiver.

This study is part of a larger project on older Pakistani women’s access to healthcare services in Norway [32] and was approved by the official data protection body at the Norwegian Centre for Research Data (Project number: 52078). All participants received written and verbal information about the study, its purpose and provided informed written consent. Participants were also informed about the rationale for doing research on Pakistani carers, i.e. the demographic significance of Pakistanis as a group in Norway and the importance of exploring access to healthcare based on previous research. The participants were also informed about the possibility to withdraw their consent at any point in the study, without any consequences. All the participant names used below are pseudonyms.

We use the term ‘residential care homes’ to refer to nursing homes, old age homes or communal living spaces for older people in need of care. The term ‘professional homecare services’ includes both home nursing care and practical assistance.

## Findings

Our analyses revealed several factors influencing family care’ perceptions of formal and informal care support, which are presented below in the following five themes: 1) caring for family in Norway as in Pakistan, 2) worries about being ‘dropped off’ at a care home, 3) concerns about being cared for by outsiders, 4) questions about what other people might say and 5) adhering to society’s expectations of a ‘good’ carer.

### Caring for family in Norway as in Pakistan

All carers reported a sense of responsibility and a desire to care for their parents/in-laws, but they had to consider what was realistic, given their busy lives, educational careers and/or professional jobs. Many felt that their mothers/mothers-in-law still had the same preferences and expectations about living with and being cared for by their children as they formerly had in Pakistan, irrespective of having migrated to Norway, as noted by a carer daughter:

They have brought this [mindset] from behind [Pakistan]. They have kept their thinking exactly the same: if it is an elderly or ill person, children should first care for them. Maybe this will change with time.

Thus, the carers believed that the concept of living together with one’s parents/in-laws largely remains amongst Pakistanis, be it locals in Pakistan or immigrants in Norway.

Some carers also pointed out how conditions in Pakistan are more favourable for caring for older people at home, as private help can be cheaply and easily hired and extended family is often nearby to help and support. They contrasted this with the situation in Norway, where there are different gender roles and where both men and women family members are employed outside the home and thus have less time for caregiving tasks. However, the lack of availability of extended support networks in Norway also made them feel more responsibility towards their parents, as described by a carer daughter:

In Pakistan, it’s like your husband is working and you are at home or if you are not at home, then your maternal or paternal aunts, or someone or other, is there who can provide care if you are going out or something. Here, it’s not like that; here, you are the only one closest, which means that if you aren’t there, nobody is there [for them].

Thus, living in Norway without extended family in the same home also evoked in carers the sense of being the *sole* carer for their older parents.

The challenges of caregiving were further exacerbated for those who also had children. Some felt restricted in doing things or going places with their children out of consideration for their older relative. For example, a carer daughter-in-law, stated:

I have children and I have to take care of them as well. If their [children’s] friends come and make a lot of noise, she would say, ‘my blood pressure is getting high’. Of course I don’t tell the children to invite their friends. I tell them that grandma can’t bear it, her head aches. My mother-in-law stays here so everyone has to come to our house. It’s obvious that one has all responsibilities then, at least I have. It’s not like everything can be done according to my wishes. If I have to go somewhere, I have to ask at home first. And if … we want to go for a holiday with children, we take her along. But she says that now she can’t travel much.

### Worries about being ‘dropped off’ at a residential care home

For the carers interviewed, thoughts about transitioning their parent to a residential care home brought up feelings of guilt, as they reflected on their parents’ care expectations and worries of being simply ‘dropped off’ at a care home in future. A carer daughter noted her parents’ unpreparedness and said, ‘I don’t think my parents will ever be prepared for a situation like that; in fact, they keep saying “do not do this to us”’.

Another carer daughter expressed how her parents perceived residential care homes as the last resort, stating the following:

[T]hey have a big fear of, ‘don’t send us there at all!’ [They say,] ‘It’s better that we pass away before that’. They have a problem with this here [in Norway], too. So I can imagine that, with time, uncles and aunts would have problems with this. I mean, to live somewhere away from home, they will have problems, so just thinking of residential care homes scares us. We will do whatever we can ourselves.

Thus, parents’ fears of being sent to residential care homes also made carer feel concerned about this transition and strengthened their resolve to take on the responsibility of providing informal care themselves as much as possible. Although a few participants reflected on the possibility that a better quality of care might be provided in professional care homes than could be provided at home, they still felt uncomfortable about the idea. For example, a carer stated her own preference for having her parents live in her own home and preferred ‘to have them in front of [her] eyes’. She believed that providing care at home would allow her to spend more time with her parents, instead of visiting them for a few hours each day or week at a care home.

While positive perceptions about the quality of care at care homes did not seem to influence carers’ preferences for care, carers also did not discount the challenges of caregiving. For instance, of this tension between parents’ wishes and carers’ abilities, one carer stated, ‘One or the other would have to make the sacrifice. I don’t think we will send them to a residential care home’.

Thus, carers expressed mixed feelings of responsibility, as well as ideas of having to make a sacrifice, pointing to their own discomfort with the idea of residential care homes. A few carers, however, took into consideration the practical challenges of caregiving and reflected on when a formal care option might be considered. One carer daughter stated the following:

[I]t also depends on how much time you can give them; if you can’t give them any time and they are close to 80 to 90 years of age, then it’s obvious that one will have to think of something for them. But then I think parents can’t decide this—children should step in.

It can be seen in this quote that the carer (in this case, a daughter) asserted the involvement of children in deciding the course of future care for parents. However, the same view was not shared by carers who were daughters-in-law, despite them sharing similar concerns about older family members being sent to residential care homes. Carer daughters-in-law, unlike daughters, expressed that they would not be the ones to decide whether to transition their mothers-in-law to residential care homes, given the relationship they shared with their older family members. For example, a carer daughter-in-law noted the following:

I think then her children would decide. Obviously, I would share my opinion, but I can’t say that this is what you should do. .. because, since I live with her, obviously it’s my responsibility. Sometimes I think about it, but then I tell myself that, when the time comes, we will see. They [the government] give a lot of help to people here.

Thus, while concerns about older mothers/mothers-in-law were shared by both daughters and daughters-in-law, the latter did not think they could be very involved in decision making regarding future care, despite perceiving the responsibility of caregiving to a greater degree than did daughters.

### Concerns of being cared for by others at home

Professional homecare services were not an optimal alternative to informal care for most of the carers either, despite the fact that such services could eliminate older parents’ concerns about being simply ‘dropped off’ at a care home. Carers mentioned their responsibility of reciprocal caregiving as integral to their preference for informal care, and a few expressed that they did not want anyone else to take care of their parent in future. For example, a carer daughter stated, ‘We do not want anyone else to take care of mother in future. When we were children, they took care of us; now it is our turn’.

Carers also reflected on their parents’ discomfort with the idea of being taken care of by ‘outsiders’, even in their own homes. For example, a daughter-in-law described how her mother-in-law might perceive this, noting the following:

I do think that in future maybe if something happens, she might need home care. .. because it has happened with many of my friends.. .. Many [mothers-in-law] would not even let outsiders touch them.. .. She may agree to it because in her heart she knows how much I can take care of her. But I think it would be difficult [for her to agree].

A carer daughter also described her mother’s similar discomfort with using professional homecare services:

My mother refused it, saying that ‘my heart doesn’t want someone else to come to my house for this’. Now, we get it for my father too, but initially, they found it very difficult to adjust, as different people used to come. When they feel that someone is staring at them, they can’t really be themselves.

She further described that she even tried to convince her mother to use a homecare service by telling her about the possibility of having a Pakistani homecare professional, but her mother still did not feel comfortable with it. However, as the situation worsened, they had to accept the use of homecare services, despite her parents’ reservations.

### Questions about what other people will say

While the preference for informal caregiving mainly stemmed from feelings and beliefs about reciprocal care and children’s responsibility to their parents in Norway, there also existed feelings of being judged by the Pakistani community if formal care services were to be used.

A carer daughter described the larger community’s perspective and how, while growing up, she used to hear about children ‘abandoning’ their parents:

Then, people used to say ‘Haiii! See what they [the children] have done!’ They might be thinking that they [the children] are getting rid of them [the parents], that they don’t want to care; but they don’t understand how much trouble they [the children] are facing—they have a job and a responsibility to manage their home.

Another carer daughter told of her dilemma when considering whether to transition her older parent to a residential care home, being torn between the ‘talk’ of the community and her own preferences and caregiving constraints. She noted, ‘that is there. .. people will talk. Among our *ger-mulkis* [immigrants], this issue is there.. .. We don’t want to do this either, but when constraints come. ..”

It should be noted that the fear of judgement from the community was not only associated with how the children might be perceived by the community but also how the parents might be perceived, as described a daughter:

They [the parents] think, ‘What will people say?’ I mean acquaintances, family, friends—it can be anyone. This also happens a lot. For parents and for children as well. Then they feel a lot of disappointment, thinking, ‘That person thought this about my children? That we haven’t given proper values to them?’ . .. That’s why they are not accepting it right now.

In light of judgement from the community, professional homecare services were also considered to be problematic, as noted by a carer: ‘Maybe they will say things like, “They are not taking care of their parents. They are [forced] to take on help”.. .. Maybe because of these things, we may not take on help’.

Thus, some carers felt that they may decline the use of professional homecare services due to fear of social condemnation. This, however, did not mean that they could accept compensation in lieu of professional homecare services, as regulated by law. A carer daughter explained the following:

Then also people will talk, like, ‘See? They are doing this to get money!’ Then it’s obvious: we will have to take on professional home care. Because people would think that we are doing this for money, it is better that we accept professional home care if we want to escape people’s gossip. ‘What will people say?’ This thing defeats us on every issue.

A few also perceived taking compensation for care as contradictory to what a Pakistani would do. For example, a carer daughter pointed out that she would never take such compensation but that her brother might, ‘because he doesn’t think like us; he is more like a Norwegian’.

Despite the potential to be gossiped about by others, most carer daughters still felt that it is acceptable to take compensation for informal care in lieu of using professional homecare services. However, daughters-in-law perceived it as much less socially acceptable, as explained by one:

[M]any of our people think this way. .. but it’s like the husband’s family members would say, ‘She has kept her for money’. I have heard from many people that. .. they would not get professional home care for money and [would instead] take care of them themselves.. .. [S]ometimes it’s also difficult to take care.. .. It’s one thing to take care of a child, but it’s another thing to take care of an older person. It’s stressful for us.

Another daughter-in-law echoed similar perceptions and reiterated that decisions related to accepting compensation for care would also depend on the husband, despite the daughter-in-law being the primary carer:

I think my husband would say, ‘Leave your work and take care of her. Leave the money, etc.’. Otherwise, people might say, ‘She takes money to take care of her. [That’s why she] has kept her at home’, and others might say, ‘Yes, her relative has become ill and she has become rich’. . .. You know, the type of women who stay at home and don’t work. .. all those people say it’s right that she gets money now and she takes care of her. .. as it’s a mentally difficult task to take care of someone. And if it’s a mother-in-law, you can’t say everything directly … This is what happens in a husband’s family.

### Adhering to the society’s expectations of a ‘good’ carer

Some carers believed that the practice and value of taking care of older parents is highly regarded by ethnic Norwegians. A carer daughter noted the following:

These Norwegians, they appreciate this thing a lot that we *ger-mulkis* [immigrants] take a lot of care of our parents. .. that, when they get old, we don’t send them to a residential care home; we care for them.

When asked how they felt this was appreciated by ethnic Norwegians, a carer daughter explained the following:

[W]hen mother was in hospital, we used to go there every day to see her and bring her food, etc. So the white people who were staying there long-term started telling their own stories, saying nobody comes to see them—only at Christmas or on other [holidays].. .. Then we understood that they long for this. .. for someone close to them to come and meet with them. Even doctors say that *ger-mulki* [immigrants] are very good at such things; they care for their elderly a lot.

Some also pointed out that Norwegians are often surprised that they (i.e., Pakistani people) do so much for their parents, while the Pakistani carers simply see this as fulfilling their responsibility.

## Discussion

The increase in the older population of the Pakistani community in Norway raises the question of their need for formal and informal (i.e., unpaid) care. The aim of this article was to explore female carers’ views on future care for their older relatives. One of the most striking findings is that nearly all the participants held the view that they would be responsible for their older family members’ care, even though formal care options may be available. Their older relatives’ wishes of living with their children and receiving care from them seems to be an expectation that the participants anticipated and felt obliged to accommodate. Traditional expectations of filial piety tend to be strongly rooted in many immigrant communities [33–35]. A study of Asian Americans showed, for example, that providing care for family members when they become old is often seen as a ‘natural’ obligation [34]. Filial responsibility is also considered, as suggested in a study about Asian family carers living in Canada, to be a cultural obligation that provides psychological rewards and personal growth [36].

Although participants in the current study generally saw providing care for their older relatives to be a duty, participants shared the view that such expectations were hard to reconcile with their life in Norway. One of the reasons for this seems to be that older relatives’ values and norms relating to family relationships mirror those that are common in their countries of origin [19] but often differ from those of their younger carers—who are more accustomed to the migrated countries’ norms [37, 38]. Therefore, the older generation’s expectations of care tend to define the norms within the immigrant communities, but these norms are often outdated even in their countries of origin [33]. Yet these norms seem to strongly inform how family carers view their ability to manage their responsibility to provide future care to older relatives.

One of the difficulties participants in our study anticipated was the concern about what other people might say if they were to choose care options for their older relatives other than family care. Their fear of breaking their communities’ norms of filial piety seemed to be a manifestation of the fear of social exclusion. Sanctioning members of a community for not living up to shared ideals of filial piety is not uncommon. In a study of Turkish immigrants in Belgium, Tavernier and Draulans (2018) contended that social pressure to conform to traditional family caregiving ideals occurred. Resisting or failing to fulfil the expectations of the community would bring about social exclusion and feelings of shame and guilt on the older immigrants’ children [33]. The pressure to care for older relatives may therefore lead to postponing or declining to seek professional care [39], as carers themselves may perceive the act of seeking external support to be ‘relinquishing their caregiving responsibility’ [40]. Social pressure that stems from idealised notions of care, which are more or less detached from the new reality in the host country, makes the use of professional care services less attractive to family carers.

Still, traditional views of filial piety, according to the family carers’ perceptions, do not entirely determine the use of or access to healthcare services of their older relatives. This seems to be in line with the assertion of Levesque et al. [41] that access to healthcare is contingent on the cultural expectations of clients as well as the characteristics of healthcare providers. Participants in our study seemed to worry about whether their older relatives would be comfortable with the available healthcare options. They were concerned that their older relatives would feel as if they were being ‘dumped’ at a care home. They worried that their relatives would feel lonely in a residential care home and that they would not be able to visit their relatives as often as they would like to. With regard to professional homecare services, participants were worried about their parents’ inhibitions and discomfort with being cared for by ‘outsiders’, even if the homecare professionals they hired were Pakistani.

The literature indicates that many older people with a migration background are sceptical about care institutions [42]. Studies of older Pakistani immigrants in Norway found that they are often afraid that inadequate support is provided in homecare services and that they will become detached from their family if they choose to live in such an institution [28, 43]. Meanwhile, professional carers—as Berdai Chaouni and De Donder [39] pointed out—are often unaware that immigrants often avoid formal care because of their perception or anticipation that the care provided will be culturally or religiously insensitive.

The family carers in the present study also gave accounts of Norwegians praising how well immigrants care for their parents when they get old and praising them for not sending their parents to a residential care home. Although caring for older relatives is admirable, it would be naïve to explain such differences between the majority population and immigrants solely based on culture. It is therefore worth looking at the structural underpinnings that make such filial support, to say the least, impractical.

As is the case in many high-cost countries, Norwegian society is based on families having two household incomes, which makes full-time childrearing while also caring for older relatives simply not feasible. Due to the traditional norms of caregiving, women from South Asian communities can face great role conflict in their attempts to juggle occupational demands and the demands of caregiving [44]. Nergård’s [45] study on the topic of expectations about old age in three immigrant groups, including Norwegian–Pakistani women aged 26–40, found that women were willing to stretch themselves far in order to ensure proper caregiving within the family. While they expressed negative attitudes towards nursing homes, most of the women did not have any reservations about using services aimed at relieving the household of some tasks. The norm of family members acting as carers is increasingly becoming difficult to uphold due to changes in the family structure, as well as conflicting responsibilities and roles [46]. Although idealised perceptions of caring for older people can be found within immigrant communities [33], it is important not to yield to assumptions that children from a immigrant background will always want to ‘take care of their own’, as this can lead to a lack of attention being given to the adaptation of healthcare services for older immigrants and their families [42].

A worldwide demographic tendency is that women, on average, have a longer lifespan than do men [47]. Since the 1960s, immigrant men in Europe have been assigned jobs that involve hard and manual labour and have therefore been more exposed to health hazards [48]. Pakistani men in Norway have similar work trajectories [26]. From a gender perspective, this means that immigrant men are most likely to be in need of informal and professional care early in old age. At the same time, due to gender norms about caregiving in immigrant communities [33, 34, 36], women end up caring for both men and women, often conflicting with childrearing responsibilities. This especially affects daughters and daughters-in-law, who often, regardless of competing responsibilities, are expected to provide care to their relatives [34]. This is exacerbated by norms that dictate, for example, that being compensated for the care provided is not consistent with genuinely caring, as pointed out by participants in our study. The participants in our study, all being women, also had the perception that they would have to make sacrifices to care for their relatives and that it would not be a realistic option for them to send their relatives to a care home.

According to Levesque et al. [41], the ability to seek out healthcare services relates to people’s autonomy and capacity to choose, based on their knowledge of the care options available and their eligibility rights. In the study, they refer to an example of immigrant women being discouraged from seeking professional help due to, among other things, discrimination. This is especially true for daughters-in-law, as they—unlike a recipient’s own children—traditionally would not be in a position to make decisions about using professional care homes. We found that daughters-in-law did not seem to play a major role in decision making regarding care options, including the decision to receive compensation for the care they provide; however, because they still carry the responsibility to fulfil the choices that are made, they often face a moral dilemma. This dilemma becomes clear when their care for older relatives coincides with their need to care for their children, a notion known as “sandwich caregiving” [49]; they often have to choose between financial security (e.g., going back to work) or social inclusion (as they would be excluded for ‘neglecting’ their caregiving responsibilities) [33]. Such dilemmas illustrate the need for culturally sensitive healthcare provision for older immigrants and support for family carers.

It is therefore important to consider whether healthcare services currently meet the needs of immigrant families and whether the way they are organised make them accessible [41]. Practical challenges such as language barriers and insensitive approaches have been mentioned in previous studies of immigrant populations, including in Norway [36, 50]. Healthcare professionals may even be aware of immigrants’ underutilisation of these services, but they often assume that immigrants will not choose formal care and thus do not target these groups systematically. Such attitudes from healthcare providers may lead immigrant families to consider alternative care options, such as the use of unofficial domestic helpers and care marriages [39]. Although carers in our study expressed their relatives’ concerns of being cared for by ‘outsiders’, they nevertheless anticipated their own challenges of informal caregiving. Thus, meeting older immigrants’ need for culturally sensitive care, both through political action and actual organisational changes in healthcare structures, may improve immigrants’ access to care and make the choice of formal care more realistic. Our findings also revealed carers’ own desire to care for their older relatives even though they believed that the Pakistani norms of family caregiving did not reconcile with life in Norway. Therefore, considerations about access to formal care also need to take into account traditional values of caregiving, not in order to reify them but to understand and even challenge them. This is even more important when older immigrants wish to ‘age in place’ without alienating their relatives, as taking traditional values for granted **results** in insufficient access to formal care, which undoubtedly increases families’ care burden.

### Limitations of the study

This study sheds light on the perceptions and experiences of female Pakistani carers, a topic which has so far been insufficiently explored. While it provides rich data, there are some limitations to this study. Firstly, this study is based on data collected through interviews with carers. Therefore, we only had access to information on their perceptions and experiences. Use of other methods such as observation or focus group discussion would have potentially given data on their behaviour as well responses in a group setting, which would have further enriched the findings.

Secondly, since we had only one participant who was not born in Norway, we could not explore the difference in perceptions and experiences between carers who were born in Norway and those who were born in Pakistan. Similarly, all our participants had higher education, were employed either part time or full time or were pursuing higher education. Therefore, our findings are specific to carers with a similar educational background. Another limitation of this study relates to the interviewer’s insider position with regard to a somewhat shared culture, language and gender. While having an insider position helped in building rapport with participants, it also presented challenges of assumptions of shared understanding [49]. However, frequent discussions with co-authors from different backgrounds, locally and internationally, and writing field notes helped us to discuss understanding behind certain interpretations.

Finally, we only spoke with female carers, which reflects that caregiving duties are disproportionately distributed between men and women and that the burden of caregiving often falls on women, as previous research has indicated [17]. However, it is possible that our recruitment strategy failed to reach out to male caregivers or men who identified themselves as primary caregivers, or that women were more likely to agree to participate since the interviewer was also a woman. The snowball method we employed may also have meant that having interviewed women at the beginning, there was a greater likelihood of being referred to other women carers.

## Conclusion

This study gives a voice to female Pakistani carers and shows that traditional expectations of filial piety make informal care more appealing for immigrants. Due to gender norms, this usually means that children, and especially daughters and daughters-in-law, assume carer responsibilities. When healthcare systems lack cultural sensitivity, immigrant families and their carers have limited care options in old age, which in turn exacerbates families’ care burden. Healthcare professionals and policymakers should not assume that immigrant families will ‘take care of their own’, but instead should secure adequate support for older immigrants and their family carers. Such measures would help to tackle their support needs while they are still minimal, reduce the burden on public care services in the long term and improve the quality of life and ability to cope among family carers of older immigrants in Norway. There is, however, a need for additional research to understand various immigrant populations’ experiences and unique challenges in accessing and using health care. Attention should also be paid to issues such as the carers’ social and financial background, their relationship with the older relative, and their place of birth. Most crucially, healthcare systems should be scrutinised to identify the obstacles that may exist for older immigrant populations in accessing the care to which they are entitled.

## Data Availability

The data analysed during the current study are not publicly available due to privacy concerns but are available from the corresponding author on reasonable request.
